# Families, Parenting and Aggressive Preschoolers: A Scoping Review of Studies Examining Family Variables Related to Preschool Aggression

**DOI:** 10.3390/ijerph192315556

**Published:** 2022-11-23

**Authors:** Raúl Navarro, Elisa Larrañaga, Santiago Yubero, Beatriz Víllora

**Affiliations:** Department of Psychology, Faculty of Education and Humanities, University of Castilla-La Mancha, Avda de los Alfares, 42, 16071 Cuenca, Spain

**Keywords:** preschool aggression, childhood, aggression, family, parenting, bullying, scoping review

## Abstract

Background: A growing body of research has shown that children behave aggressively from an early age. In recent decades, such behaviour has become a focus of scientific interest, not only because of the adverse consequences of these interactions, but also because high levels of aggression, especially at an early age, may be a risk factor for the use of other forms of aggression, such as bullying, later on during their development. These behaviours are related not only to individual characteristics, but also to peer relationships, teacher behaviours, school variables, family factors and cultural influences. Method: In order to find out which family variables have been researched in relation to preschool aggression and which family variables are associated with perpetration and victimisation, a scoping review was conducted in accordance with the PRISMA guidelines. Four databases (Web of Science, Scopus, PubMed and PsycINFO) were used to map the studies published between 2000 and 2022. Results: This scoping review included 39 peer-reviewed articles from an initial sample of 2002 of them. The majority of studies looked only at perpetration behaviours. The main family variables covered in the articles concern parental behaviours, adverse childhood experiences in the family environment, and the household structural and sociodemographic characteristics. Conclusion: This scoping review shows that different factors within the family environment increase the risk of developing aggressive and victimising behaviours in the preschool setting. However, the relationship between the family variables and preschool aggression is complex, and it may be mediated by other factors such as gender, child–teacher closeness or parent–child dyads.

## 1. Introduction

Aggression has been defined as “any form of behaviour that is intended to injure someone physically or psychologically” [1]. By this definition, there are two essential components to aggressive behaviour: the intent to harm another person or persons, and this intent takes the form of harmful behaviour. Aggressive behaviour is generally classified as physical (hitting, pushing and shoving, spitting, etc.), verbal (shouting, making threats, insulting, etc.,) and relational (spreading rumours, ostracism, exclusion, etc.) [2,3]. The scientific literature considers physical and verbal aggression as a form of overt or direct aggression [4], and relational aggression is considered as a form of indirect and social aggression [5].

A significant number of studies show that boys and girls behave aggressively towards others from a very early age [6,7]. More precisely, physically aggressive behaviour can be observed in young children in early childhood, and it seems to intensify by the ages of two and three [8,9]. However, childhood physical aggression tends to decline with age and assume ever more subtle forms that are less obvious to adults [10,11]. In fact, the research suggests that children around 30 months old exhibit relational aggression in their interactions with other children [12], which is easily distinguishable from physical aggression [13].

It is unclear whether children intend to cause physical or psychological harm in infancy, although some studies indicate that children as young as three–six years old understand what it means to harm others, and so their actions should be considered to be a form of aggressive behaviour [14]. In fact, some researchers also suggest that children may intentionally harm others physically and relationally during childhood, while also using similar means to achieve some goals without meaning to cause harm. Take the example of a child forcibly grabbing a toy they like from the hand of their playmate. In this case, the main goal is to get the toy, rather than to intentionally harm the playmate [15]. The existence of objectives other than causing harm does not diminish the aggressiveness of the behaviour. Instead, we find ourselves in the realm of proactive aggression, which is born out of self-interest and is aimed at achieving some end, as opposed to reactive aggression, which is rooted in anger and seeks to cause harm [16]. Along these lines, Roseth and Pellegrini [17] found that children are already using proactive aggression in childhood to intimidate others or achieve dominance within the peer group.

Proactive aggression involves greater premeditation and intentionality rather than reactive aggression, and it more closely resembles the characteristics of school bullying [18]. There is growing discussion in the scientific literature on whether the term bullying can be used when one is researching aggressive behaviour at the preschool stage [19]. Bullying is physically, verbally or psychologically aggressive behaviour that is deliberate and repeated over time. It is further characterised by a power imbalance between the perpetrator and the victim, where the victim is or feels in a weaker position due to differences in physical strength, social skills, social status and so on [20]. Various studies suggest that bullying behaviour can exist as early as the preschool stage [21,22]. In particular, the research shows that the typical features of bullying are already evident during childhood. These include observing repetitive behaviours, identifying different roles—victim, aggressor, bystander and defender [23]—and the fact that intentionality is already present in such displays of aggression [16]. However, other researchers have noted significant differences between preschool aggressive behaviour and the later stages of schooling or adolescence, such as the absence of gender differences among the victims, role instability [24,25] or difficulties in recognising peripheral roles such as the bystander or the defender [26,27].

Irrespective of the term that is used, the above data suggest that aggression is a major issue that needs to be addressed in the early years of schooling when children begin to interact with their peers and experiment with different social behaviours. In recent decades, such behaviour has become a focus of scientific interest. This is not only due to the associated adverse consequences, but also because high levels of aggression, especially at an early age, may be a risk factor for the use of other forms of aggression at the later stages of development, such as bullying itself [27,28,29].

The early developmental factors associated with these behaviours are the subject of mounting research, with many studies examining the cognitive and emotional factors underlying these early displays of aggressive behaviour [30,31,32,33,34]. The socioecological theory [35] suggests that children’s behaviour is influenced by four structures: the individual characteristics (biological and personal background) that have an impact on children’s behaviour and social relationships; the immediate social and physical environment (where primary socialisation takes place); community-related factors (the formal and informal social institutions and structures where relationships develop); the society at large (the economic and social environment, including cultural norms). Within the immediate social and physical environment, the family context is fundamental for children’s personal, social and educational development. This is the first socialisation context in which attitudes and behaviours that will guide social behaviour in other contexts such as school are learned and reinforced [36]. The past research has shown that aggressive behaviours are associated with different family variables, such as the parents’ attitudes toward violence, the parents’ moral disengagement, a lack of family communication, an absence of emotional support, a deficient level of family cohesion, low levels of parental acceptance of their children, high levels of coercion/imposition (rules, limits, punishments, etc.), and having problematic relationships with their siblings [14,18].

Insight into how family variables relate to aggressive behaviours during the pre-primary educational stages is crucial for gaining a better understanding of the associated risk factors and protective mechanisms that can improve the prevention and intervention methods [37,38] and prevent spillover to the later educational stages [39]. In light of this need, a scoping review is an ideal way to determine the nature and volume of research, and thus, gauge the current state of knowledge in this field. This scoping review therefore aims to analyse the peer-reviewed literature on aggressive behaviours among preschool children in school settings together with associated family factors, synthesise the available evidence and outline the future research priorities.

## 2. Materials and Methods

### 2.1. Design

We conducted a scoping review of the published scientific literature on aggressive behaviour in preschool settings and the family factors linked to such behaviour. Scoping reviews provide an overview of the research that has been conducted on a specific topic, describing the body of existing work, giving an insight into how the previous studies have been conducted, identifying the variables related to the topic that is under study and highlighting the gaps in the research [40]. In the past, scoping reviews have been used to assess the available evidence on the prevalence, measurement instruments, risk factors and consequences of different forms of aggression in children and adolescents. For example, Nelson et al. [41] used this approach to identify the validated instruments that measure aggression and bullying among pre-adolescents and children. Bender et al. [42] conducted a scoping review to find out the role that is played by guns in adolescent dating violence. Srinivasan et al. [43] employed this approach to map the research on bullying among children and adolescents in low- and middle-income countries. 

The design and development of this scoping review followed the five-step methodological framework outlined by Levac et al. [44]: (1) identifying the research question, (2) identifying the relevant studies, (3) selecting the studies, (4) the charting and data analysis stage and (5) summarising and reporting the results. The screening of the studies and the summarising and reporting of the articles finally included in the review are described in line with the Preferred Reporting Items for Systematic Reviews and Meta-Analyses extension for Scoping Reviews (PRISMA-ScR) [45]. The protocol for this review was not registered in advance.

#### 2.1.1. Stage 1: Research Question

This scoping review was guided by the following question: *what is known about the family variables related to peer aggression and victimisation in preschool settings?* Specifically, we wanted to find out about the forms of preschool aggression explored by the research, the family variables analysed in relation to preschool children’s aggression, the methodology used to analyse these associations and the family variables that are most related to perpetration and victimisation in the preschool context. Prior to the review process, aggression in preschool settings was defined as any act in which a child or group of children insults, hits, socially excludes or threatens other classmates in the school. Aggression therefore includes any act of a physical, verbal or relational nature. These acts can be both reactive and proactive, and the researchers may use various terms such as aggression, victimisation, peer aggression, unjustified aggression or bullying, among others. According to the Center for Disease and Control Prevention [46] *preschoolers* are children from three–five years old. However, the age range varies across countries, and they may be as young as two years old, and some children in their last year of preschool may have reached six years old before the end of the year, so for the purposes of this review, the children of preschool age included those aged 24–72 months. With regard to the family factors, the aim was to analyse the variables associated with parenting (parenting styles, support, attachment, communication, etc.), family structure (including siblings), socioeconomic level, parental educational attainment and other variables such as the existence of family disputes, interparental violence, child abuse and parental psychopathology. 

#### 2.1.2. Stage 2: Identification of Studies

We used four bibliographic databases for the review: Web of Science (WoS), Scopus (SP), PubMed (PM) and PsycINFO (PI). The literature search was conducted in July 2022, and it was restricted to English and Spanish language articles that were published during or after 2000. The search was restricted to three dimensions with a search string for each one (see Table 1 for all of the dimensions and terms used): participants (Dimension 1), preschool aggression (Dimension 2), family context (Dimension 3). To ensure that we conducted as complete a search as possible, variants or synonyms of the established search terms were used. The search was carried out by combining the title, abstract and keywords in the four databases, and it was performed in both English and Spanish. 

#### 2.1.3. Stage 3: Study Selection

The search was conducted by the first and last authors (RN and BV, respectively), who both have experience with the previous reviews [47]. They also screened and selected the articles resulting from each database search, discussing any questionable records until an agreement was reached. Any remaining doubts or disagreements were discussed and resolved with the other authors.

The studies were included in the final analysis if they met the following criteria (see Table 2): (1) The studies had to be published in English or Spanish, in peer-reviewed journals and be quantitative or quantitative in nature. (2) The study participants had to be aged between the ages of two (24 months) and six (72 months) years old, thus spanning the second cycle of preschool education. If the studies looking at several age groups were included, only the data relating to the relevant age range (two–six years old) were reviewed. When no age range was reported, two criteria were taken into account for inclusion in the study: firstly, that the authors explicitly stated that the study was conducted in a preschool (and not a primary school or above), and secondly, that the mean age of the participants was from two–six years old with a small standard deviation. In longitudinal studies, only the information linked to the established age range was reviewed. For example, if a study looked at children aged two–six years old, and it measured the same participants at the ages of nine and fourteen, only the baseline measurement was taken into account in analysing the relationship between the aggressive behaviour and the family variables. (3) The study participants had to be from the general population, and research involving specific groups or clinical samples was excluded. (4) The articles had to analyse aggressive peer interactions in the school environment. As previous studies have shown that the parents may not be reliable as the sole sources of information on aggression outside the family [48], those that only measured the parent-reported preschool aggression and did not include the teachers’ reports, the school observation procedures or the peer nomination methods were excluded. (5) The analyses in the study had to include one or more family variables as predictor variables. If the study contained other family variables, but they were included as control variables or covariates in the analyses, only the results of the family variables that were included as predictors were reported.

**Figure 1:**
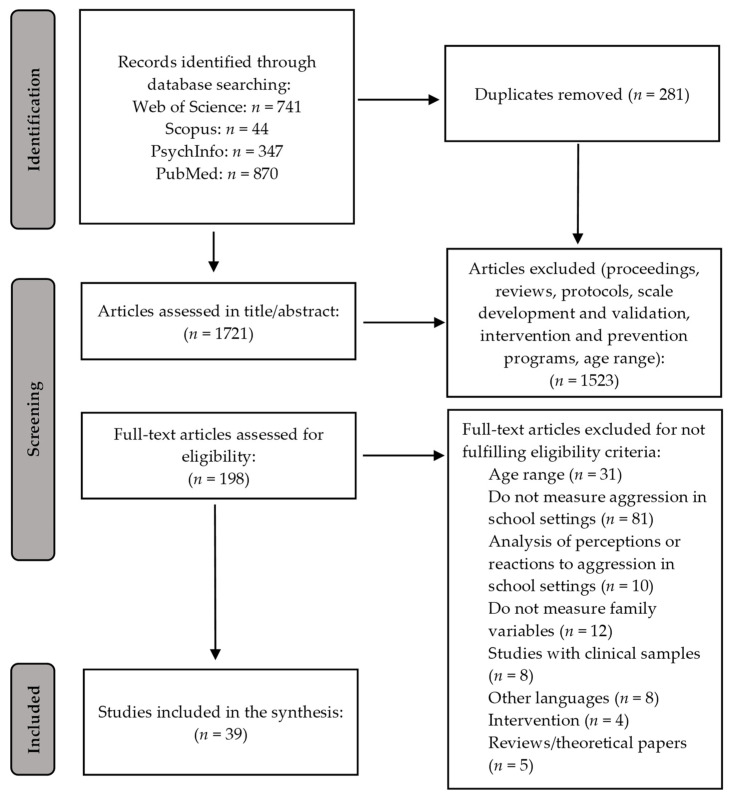
Selection procedure flow chart.

Figure 1 shows the PRISMA flow chart, illustrating the identification, screening and selection process. The search for the terms described above in all of the databases used yielded 2002 articles. A total of 281 duplicate results were eliminated, leaving 1721 for the first phase of screening via the title and abstract. In this first screening, 1523 articles were eliminated, leaving 198 articles for the full screening. A total of 159 articles were eliminated after fully screening them against the above research question and exclusion criteria. In the end, 39 articles met the inclusion criteria and were included in the review.

#### 2.1.4. Stage 4. Charting and Data Analysis

Garrad’s matrix method [49] was used to extract information from each of the studies that was included in the final selection and synthesise their findings. The matrix (see Table A1) includes the geographical location, the year of publication, the sample size and characteristics, the study objectives, the forms of preschool aggression analysed, the methodology used to measure preschool aggression, the family variables included in the analyses, the type of statistical analysis used to analyse the relationship between preschool aggression and family variables, and the main findings on this relationship. The selected articles were organised into chronological order so to better visualise the evolution of the literature on the subject that was under analysis.

## 3. Results

This section organises and summarises the results obtained in fulfilment of Stage 5 in the development of the scoping review.

### 3.1. Sample Characteristics

The studies included in the review were conducted in 2000–2022. Table A1 shows the sample characteristics (see Appendix A). Most of the study samples were from the United States (*n* = 14; 35.89%), which was followed by Turkey (*n* = 3; 7.69%), China (*n* = 2; 5.12%), Hong Kong (*n* = 2; 5.12%), Canada (*n* = 2; 5.12%), Japan (*n* = 2; 5.12%), Australia (*n* = 2; 5.12%), Taiwan (*n* = 1; 2.56%), Singapore (*n* = 1; 2.56%), South Korea, (*n* = 1; 2.56%), Egypt (*n* = 1; 2.56%), Belgium (*n* = 1; 2.56%), Netherlands (*n* = 1; 2.56%), Iran (*n* = 1; 2.56%), Russia (*n* = 1; 2.56%), Norway (*n* = 1; 2.56%), Croatia (*n* = 1; 2.56%), Spain (*n* = 1; 2.56%) and Israel (*n* = 1; 2.56%). The children ranged in age from two years and two months to six years old. No studies were found from Latin America. Most of the studies included both male and female participants (*n* = 38; 97.43%), and more than half of the studies specify the participants’ ethnicity (*n* = 20; 51.28%). The sample included predominantly White participants. Of the thirty-nine studies, five of them included Asian participants (12.82%), seven of them included Latino participants (17.94%) and seven of them included Black participants (17.94%).

### 3.2. Methodological Differences

Of the 39 international articles included in this scoping review, twenty-five of them had a cross-sectional design (64.10%), seven of them had a longitudinal design (17.94%), and seven of them had a short-term longitudinal design (17.94%). No cross-cultural studies were found.

For the most part, the informants for the data collection were children, parents and teachers (*n* = 29; 74.35%). When the parents were the informants, only one study included couples of the same gender (2.56%), and only one study (2.56%) included a peer nomination procedure. In most of the studies, a composite measure was used to analyse the aggressive behaviour (*n* = 19; 48.71%). Physical aggression (*n* = 21; 53.84%), relational aggression (*n* = 17; 43.58%) and verbal aggression (*n* = 8; 20.51%) were the most frequent forms of aggression. The most frequently analysed one was perpetration (*n* = 36; 94.87%), which was followed by victimisation (*n* = 5; 12.89%) and the combined perpetrator-victim role (*n* = 1; 2.56%). No studies were found which analysed the roles of bystander and defender.

Of the instruments used to measure preschool aggression via the teachers’ reports, the most common ones were: the “Preschool Social Behaviour Scale” [50] (*n* = 8; 20.51%), the “Social Competence and Behaviour Evaluation” scale (SCBE-30) [51] (*n* = 4; 10.25%), the “Child Behaviour Scale” (CBS) [52] (*n* = 4; 10.25%), and the teachers’ reports using items adapted and expanded from Dodge and Coie [53] (*n* = 3; 7.69%). The methods of enquiry also included naturalistic classroom observations (*n* = 4; 10.25%).

There was no overlap in the instruments used in the studies when the parents were the informants of the aggressive behaviour. The instruments were: the parents’ reports using the “Preschool Social Behaviour Scale” [50] (*n* = 1; 2.56%), the “Children’s Social Experiences measure” (CSE) [54] (*n* = 1; 2.56%), the “Children’s Social Behaviour” scale (CSB) [55] (*n* = 1; 2.56%), the “Family and Child Experiences Survey (FACES) interview” [56] (*n* = 1; 2.56%), the “Aggression Scale” developed by Shahim [57] (*n* = 1; 2.56%) and the “Strengths and Difficulties Questionnaire” (SDQ) [58] (*n* = 1; 2.56%).

In terms of the statistical analyses, 97.93% of the articles used both bivariate and multivariate analysis, 2.56% of them used bivariate analyses, 7.69% of them used mediation path analyses, 12.82% of them used moderation analyses and 5.12% of them included interaction analyses. The mediating and moderating variables included: the mothers’ depressive symptoms and the children’s social awareness, authoritative control and perceived parental rejection, child–teacher closeness, the children’s social information process, theory of mind, the aggressive decision-making process, the children’s gender and the children’s effortful control.

### 3.3. Family Variables Included in the Studies

The main family variables included in the studies were those relating to the parenting behaviours, including: psychological control (*n* = 7; 17.94%); parenting styles (*n* = 6; 15.38%); warmth and affection (*n* = 3; 7.69%); physical coercion (*n* = 3; 7.69%); neglect/rejection (*n* = 2; 5.12%); parental self-efficacy (*n* = 2; 5.12%); attachment (*n* = 4; 10.25%); parental values (*n* = 1; 2.56%); social coaching quality (*n* = 1; 2.56%); positive and negative emotion (*n* = 1; 2.56%); emotional regulation (*n* = 1; 2.56%).

Another group of studies looked at the adverse childhood experiences (ACEs; *n* = 7; 17.94%), including: physical abuse (*n* = 5; 12.82%); exposure to violence (*n* = 2; 5.12%); chronic illness within the family (*n* = 1; 2.56%); the death of a family member (*n* = 1; 2.56%); psychopathology (*n* = 6; 15.38%); parent–child conflict (*n* = 3; 7.69%); family stress (*n* = 1; 2.56%); history of maternal smoking during pregnancy (*n* = 1; 2.56%); problems during pregnancy (*n* = 1; 2.56%).

A number of studies also analysed the sociodemographic variables, including: family structure (*n* = 6; 15.38%); education (*n* = 4; 10.25%); occupation (*n* = 3; 7.69%); socioeconomic situation (*n* = 3; 7.69%); unemployment (*n* = 1; 2.56%); the number of siblings (*n* = 3; 7.69%).

Other variables analysed were: the parents’ working hours on weekdays and weekends (*n* = 1; 2.56%), the number of working days (*n* = 1; 2.56%), the length of time spent with the child and the activities they do together (*n* = 1; 2.56%).

There is great variability in the instruments used with the most common ones being: the “Parenting Practices Questionnaire” (PPQ) [59] (*n* = 3; 7.69%) for assessing parental styles, the “Parenting Sense of Competence Scale” (PSOC) [60] (*n* = 2; 5.12%) for measuring parenting self-efficacy, and the “Psychological Control Measure” [61] (*n* = 2; 5.12%) and “Parental Psychological Control” (PPC) [62] (*n* = 2; 5.12%) for measuring psychological control.

### 3.4. Primary Analysis

The results are organised according to the family variables analysed in relation to perpetration and victimisation. Table A1 summarises the main findings of each study on the relationship between preschool aggression and family variables.

#### 3.4.1. Sociodemographic Variables

##### Number of Siblings

Three cross-sectional studies (7.69%) examined the relationship between the number of siblings and preschool aggression. No studies found a positive association between the number of siblings and the perpetration of preschool aggression [63,64,65]. The search did not yield any studies researching the association between the number of siblings and victimisation.

##### Family Structure

Six cross-sectional studies (15.38%) examined the relationship between a single-parent status and preschool aggression. Three of these found no positive association with perpetration [63,64,66]. Among the studies founding positive associations, Honing and Su [67] found differences by gender. In particular, the children whose custodial parent was of the same gender received lower scores on the aggression measure from their teachers than the children whose custodial parent was of a different gender. Jansen et al. [68] found that single parenthood increased the risk of children being perpetrators and perpetrator/victims of preschool aggression. In terms of the type of aggression, Baker et al. [69] found that children in single-parent families have a higher risk of perpetrating proactive relational, reactive relational and reactive physical aggression. In the same vein, Meysamie et al. [65] found an association between single parenthood and relational aggression. Only one study examined the associations between the family structure and victimization [68], and none of them found a positive relationship.

##### Socioeconomic Level

Three cross-sectional and longitudinal studies (7.69%) examined the relationship between the socioeconomic level and aggression in preschool. The studies reveal a positive association between perpetration and low economic status, although there are differences depending on the type of aggression. Jansen et al. [68] reported that socioeconomically disadvantaged children were more at risk of being perpetrators or perpetrator/victims for all forms of aggression. Baker et al. [70] found that children with a low socioeconomic status scored higher for relational aggression but not for physical aggression when they were compared to the children with a high socioeconomic status.

In terms of victimisation, there is no clear picture. Navarro et al. [71] reported that the risk of peer victimisation increases for the children with a low or low-middle socioeconomic status. However, the same relationship was not found by Jansen et al. [68].

##### Level of Educational Attainment

Four cross-sectional studies (10.25%) examined the relationship between parental education attainment and preschool aggression. The results reveal a positive association between perpetration and low parental educational attainment. For example, low parental educational attainment increased the risk of the children being perpetrators or perpetrator/victims [68]. Along the same lines, less educated parents were more likely to have preschool children exhibiting both proactive and reactive aggressive behaviours [66]. With regard to the type of aggression, Meysamie et al. [65] found that a maternal educational attainment below undergraduate level was associated with parent-reported verbal aggression. However, paternal educational attainment was not associated with physical or relational aggression. Amin et al. [72] found lower aggression scores among the children whose mothers were university educated (3.8 vs. 4.8), but these results did not achieve a statistical significance. Only one study looked at the association with victimisation, with Jansen et al. [68] finding that of all of the socioeconomic indicators, only low parental educational attainment was associated with victimisation.

##### Parental Occupation

Three cross-sectional studies (7.69%) examined the relationship between occupation and preschool aggression. Most of the studies show a positive association with perpetration. The children of working mothers show more aggressive behaviour than the children of stay-at-home mothers [65,72]. However, Katsurada [63] does not find the same association. No studies have been found analysing the association between parental occupation and victimisation.

##### Unemployment

Only one cross-sectional study (2.56%) examined the relationship between unemployment and preschool aggression. Family unemployment was associated with an increased likelihood of being a perpetrator or perpetrator/victim in preschool [68]. However, the same association was not found for victimisation.

#### 3.4.2. Adverse Childhood Experiences

As outlined below, most of the studies have examined one single adverse childhood experience in relation to preschool aggression. However, one of the studies that was reviewed (2.56%) examined the cumulative effect of experiencing several adverse childhood experiences. Jiménez et al. [73] examined the longitudinal relationship between preschool aggression and different adverse childhood experiences including child abuse, parental substance use, incarceration and the caregiver being treated violently. Their results showed that an experience of three or more ACEs was associated with an increase in the perpetration of aggressive behaviours.

##### Exposure to Violence

Two short-term longitudinal studies (5.12%) examined the relationship between exposure to violence and preschool aggression. The children that were reported to have witnessed violence within the family were more likely to attribute hostile intentions to their peers, to respond aggressively and to deem socially unacceptable responses appropriate [74]. Along the same lines, an older sibling’s relational and physical aggression was predictive of a younger sibling’s relational and physical aggression towards their peers [11]. No studies have been found which examine the association between exposure to violence and victimisation in preschool.

##### Parent–Child Conflict

Three studies (7.69%), one longitudinal one [75] and two cross-sectional ones [76,77], examined the relationship between parent–child conflict and preschool aggression, yielding mixed results. The longitudinal study showed that children with fewer conflicting family relationships during preschool display less aggressive behaviours in school settings [75]. Ostrov and Bishop [76] found a positive association with relational aggression, but not with physical aggression. However, Farver et al. [77] did not find this association for any types of aggression. For victimisation, no studies examining these relationships have been found.

##### Physical Abuse

Five cross-sectional and longitudinal studies (12.82%) examined the relationship between childhood physical abuse and preschool aggression. All of the longitudinal [74,78,79] and cross-sectional studies [80] found a positive association between physical abuse and perpetration. Some of these studies have found differences depending on the gender of the children and their parents. For example, Ngee Sim and Ping Ong [80] unexpectedly found that paternal spanking was associated with aggression regardless of the child’s gender, while maternal spanking was associated with child aggression only when the child’s perceived rejection value was low. Moreover, maternal spanking was associated with the sons’ aggression, while paternal spanking was associated with the daughters’ aggression only when the rejection value was low. No significant associations have been found between physical abuse and victimisation [71].

##### Parental Criminality

One cross-sectional study (2.56%) examined the relationship between parental criminality and preschool aggression. Having a parent with a criminal record was significantly associated with high levels of child aggression. The association was stronger when was parents were involved in violent and frequent offending [81]. No studies have been found looking at the association between parental criminality and preschool victimisation.

##### Psychopathology

Six studies (15.38%) examined the relationship between mental illness and preschool aggression. In terms of perpetration, the longitudinal studies suggest a positive association [78,81,82], although they find that maternal mental illness plays a greater role than paternal mental illness [81,82]. However, the picture is somewhat less clear for the cross-sectional studies. Jung et al. [83] found that the children of mothers with elevated depressive symptoms were rated by their mothers as being more aggressive than the children of mothers with non-elevated depressive symptoms. However, the teachers reported no significant differences. Farver et al. [77] found no direct association between the mothers’ depressive symptoms and their children’s aggressive behaviour. In relation to victimisation, only one study (2.56%) analysed the association between parental psychopathology and being a victim of aggression in the school environment, but none of them found any significant associations [71].

##### Chronic Disease within the Family

A single cross-sectional study (2.56%) examined the relationship between chronic illness within the family and preschool aggression, reporting that chronic illness within the family is associated with verbal and relational aggression [65]. No studies have been found which looked at the association between chronic illness within the family and victimisation.

##### Death of a Family Member

One cross-sectional study (2.56%) looked at the association between the death of a family member and preschool aggression, finding a positive relationship between the death of a family member and the perpetration of verbal aggression. However, the same association was not found for physical or relational aggression [65]. No studies have been found which examine the association between the death of a family member and victimisation.

##### Problems during Pregnancy

A single longitudinal study (2.56%) examined the association between being a victim of preschool aggression and problems during pregnancy, but none of them found a significant association [71]. No studies were found that looked at the association between perpetration and problems during pregnancy.

##### History of Maternal Smoking during Pregnancy

One cross-sectional study (2.56%) analysed the maternal smoking history during pregnancy in relation to preschool aggression, but it did not find any significant association [65]. No studies were found examining the same relationship for victimisation.

##### Family Stress

Only one of the reviewed studies (2.56%) examined the relationship between family stress and preschool aggression. DeMulder et al. [84] found that higher levels of family stress were only significantly related to anger/aggression in boys. No studies were found examining this same relationship for victimisation.

#### 3.4.3. Parenting Practices

##### Parental Styles

Six studies (15.38%), two longitudinal ones [71,85] and four cross-sectional ones [66,80,86,87], examined the relationship between parenting styles and preschool aggression. With regard to perpetration, Casas et al. [86] found that authoritative and permissive parenting styles were positively related to the children’s relational aggression, and an authoritative parenting style was associated with lower levels of physical aggression in children. Jia et al. [66] found that hostile/coercive parenting was an independent risk factor for both proactive and reactive aggression in the preschool environment. However, other studies have not found a significant direct or indirect association between parenting styles and aggressive behaviour in preschoolers [80,85,87]. In the case of victimisation, only one study analysed this relationship. The results showed that the parental practices characterised by lower norms at the age of three increased the risk of the parents and teachers reporting persistent victimisation at the ages of four and five [71].

##### Physical Coercion

Three studies (7.69%), one longitudinal one [88] and two cross-sectional ones [89,90], examined the relationship between physical coercion in childhood and preschool aggression. All of them found a positive association with perpetration, though there were differences depending on the gender of the children and their parents. For example, Nelson et al. [89] found a positive association between physical coercion and aggressive behaviour, but only in boys. With regard to the parent–child dyad, Lau et al. [88] found differences depending on the parent. Maternal physical coercion was longitudinally associated with both physical and relational aggression. However, paternal physical coercion was longitudinally correlated only with physical, but not relational aggression. As for preschool victimisation, no significant associations with physical aggression have been found [90].

##### Psychological Control

Seven cross-sectional and longitudinal studies (17.94%) examined the relationship between psychological control and preschool aggression. Most of the longitudinal [86,91] and cross-sectional research [61,89] reveals a positive association with the perpetration of both physical and relational aggression. Differences were found depending on the dimensions that were studied within psychological control and the parent–child dyads that were analysed. For example, Nelson et al. [61] found that all of the dimensions of psychological control (shaming/disappointment, constraining verbal expression, withdrawal of love and guilt induction) were associated with physical and relational aggression, with the exception of invalidating feelings, albeit predominantly in same-gender parent–child dyads. However, in a longitudinal study, Lau et al. [92] found that neither maternal nor paternal psychological control was associated with either physical or relational aggression. No studies were found which examined the same relationship with victimisation.

##### Neglect/Rejection

One cross-sectional study (2.56%) examined the relationship between neglect/rejection and preschool aggression. Shim and Kim [90] found that parental neglect/rejection increased the likelihood of peer victimisation in preschool. No studies have analysed the direct relationships between parental rejection and perpetration.

##### Warmth and Affection

Three studies (7.69%), two cross-sectional ones [63,90] and one longitudinal ones [79], examined the relationship between warmth and preschool aggression, with mixed results. Low warmth/responsiveness was the best precursor of later peer aggression in the children with low levels of theory of mind [79]. In the same vein, Katsurada [63] found that children who had more positive physical contact with their parents at home were less likely to be rated as extremely aggressive by their teachers. However, when both of the parents’ physical affection scores were simultaneously inserted into the equation as independent variables, only the father’s score was significant. With regard to victimisation, Shim and Kim [90] found no association between warmth and victimisation in preschool children.

##### Attachment

Four studies (10.25%) examined the relationship between attachment and preschool aggression. In the case of perpetration, the longitudinal [85] and cross-sectional [84,86,93] analyses found a positive relationship between an insecure attachment and aggressive preschoolers. Paschall et al. [85] found that children exposed to detached parenting, which is characterised by the highest level of detachment, a moderate level of negative regard and the lowest level of supportiveness, showed higher levels of aggression at 36 months than the children who were exposed to sensitive parenting. However, there were no significant differences in the aggression levels between the children exposed to detached as opposed to harsh parenting, or between the children exposed to sensitive as opposed to harsh parenting. Casas et al. [86] found that the association between insecure attachment and relational and physical aggression varied with the gender composition of the parent–child dyad. Parent–child closeness moderates the relationship between a lower quality of attachment and aggressive behaviour [93]. No studies have been found which examine this same relationship with victimisation.

##### Parenting Self-Efficacy

Two cross-sectional studies (5.12%) examined the relationship between parental self-efficacy and preschool aggression. These studies found that low perceived parental self-efficacy among mothers was significantly associated with higher levels of aggression and peer victimisation in the school environment [94,95]. This relationship was stronger between parental self-efficacy and peer victimisation [95].

##### Parental Values (Individualism, Collectivism and Verticalism)

A single cross-sectional study (2.56%) involved the relationship between parental values and preschool aggression. The combination of maternal individualistic and collectivistic values was associated with a higher social competence in children, and thus, less aggressive behaviour. This association was not significant for fathers [96]. No studies were found examining this same relationship with victimisation.

##### Social Coaching Qualities (Elaboration, Emotion References and Rule Violation)

A single short-term longitudinal study (2.56%) looked at the social coaching qualities in relation to preschool aggression. After controlling for physical aggression, the children whose mothers used medium or high levels of emotion-focused, elaborative social coaching about relational conflict were less relationally aggressive in the following year. However, the maternal use of rule violation did not explain the individual differences in the children’s use of relational aggression, nor did it moderate the association between time 1 and time 2 relational aggression [97]. No studies examining this relationship have been found for victimisation.

##### Other Emotional Variables

Two studies (5.12%), one cross-sectional one [98] and one longitudinal one [88], examined the relationship between the caregivers’ emotional cues and preschool aggression. Mizokawa and Hamana [98] found a positive association, albeit with differences depending on the gender of the children and their parents. Boys with higher levels of theory of mind and mothers with high negative emotional expression show higher relational aggression in the preschool environment. No effect was found for girls. Contrary to previous studies, Lau and Williams [88] unexpectedly found that higher levels of reappraisal and lower levels of maternal suppression were associated with greater child relational aggression. The authors explained that these unexpected results could be mostly due to cultural factors. They did not find a significant association with paternal emotional regulation. No studies examining these same associations have been found for victimisation.

#### 3.4.4. Other Factors

##### Daily Working Hours and Days of Work

Of the studies that are included in the review, one cross-sectional study examined the association between the fathers’ daily and weekly working hours and preschool aggression [99]. The results indicate that the duration of the fathers’ daily and weekly work and the number of days worked per week were significantly associated with their children’s levels of aggression. The number of hours worked per day was the variable most strongly related to aggressive behaviour. The more hours that they worked, the higher the levels of aggression were among the five and six-year-olds in the school setting. No studies were found analysing this same association for victimisation.

##### Time Spent with the Child

Güngör et al. [99] analysed the association between the time spent with the children and preschool aggression. They found that not spending time with children increases the likelihood of the children behaving aggressively towards their classmates. No studies have been found examining the same association for victimisation.

## 4. Discussion

In recent decades, there has been growing concern to analyse the factors that influence the development of aggressive behaviour in the classroom. The socioecological theory [35] points to explanatory factors in the family environment that could be responsible for the children developing aggressive and victimising behaviours in the school setting. On the basis of this premise, this review aimed to examine the scientific literature in order to answer the following question: *what is known about the family variables related to peer aggression and victimisation in preschool settings?* This scoping review includes 39 peer-reviewed articles from an initial sample of 2002 of them, with the main conclusions of each study having been extracted and summarised.

The review of the studies detailed here indicates that the family variables associated with the perpetration of preschool aggression. Positive associations have been found with: single parenthood [65,68,69]; a low economic level [68,69]; a low parental educational attainment [65,66,68]; unemployment [68]; working mothers [65,72]; exposure to violence [11,74]; parent–child conflict [75,76]; physical abuse [74,78,79,80]; parental criminality [81]; mental illness [78,81,82,83]; chronic disease within the family [65]; the death of a family member [65]; family stress [84]; authoritative and permissive parenting [86]; hostile/coercive parenting [66]; physical coercion [88,89,90]; psychological control [61,86,89,90]; a low level of warmth in children with low levels of theory of mind [79]; insecure attachment [84,85,86,93]; low parental perceived self-efficacy [94,95]; low levels of elaborative, emotion-focused social coaching [97]; high maternal negative emotional expression [98]; higher levels of reappraisal and lower levels of suppression [92]; the duration of the fathers’ daily and weekly working patterns [99]. Negative associations have been found with positive physical contact [63]; custody with same-sex parents [67]; maternal reports of the combination of individualistic and collectivistic values [96].

Overall, the studies reviewed suggest that negative parental behaviours characterised by physical coercion, insecure attachment, low parental perceived self-efficacy and punitive styles are those which are most commonly associated with perpetration. Along the same lines, exposure to traumatic situations and violence affecting the family environment (exposure to violence, physical abuse, parental criminality, chronic illness within the family, the death of a family member, family stress and psychopathology) make a significant contribution to the development of peer aggression at the preschool age. However, there are inconsistent results for other factors such as single parenthood and parental occupation, psychological control, psychopathology and authoritative parenting.

Furthermore, the studies show some sociodemographic factors which increase the risk of perpetration in preschools. The results of this review indicate a positive association between unemployment, low parental educational attainment and economic status with the perpetration of preschool aggression.

Little is known about the family factors and their relationship to victimisation in preschool children. The research has found a significant association with the family variables such as low or low-middle socioeconomic status [71]; low educational attainment [78]; parental practices characterised by lower norms [71]; parental neglect/rejection [90]; low parental perceived self-efficacy [94,95]. These factors may affect the development of mature behaviours such as autonomy, assertiveness and self-confidence [90], as well as competence in conflict resolution with peers and a lack of coping strategies for aggressive behaviour [68,71], which may increase their vulnerability to victimisation. However, it is difficult to draw clear conclusions due to the limited analysis of the family factors in relation to victimisation. Further research is needed to explore the impact of family variables on preschool victimisation.

As they are put in this way, the existing evidence lends support to different theoretical frameworks, such as Bowlby’s attachment theory [100], the social learning theory and the intergenerational transmission of violence [101], and the family-relational schema [102]. These theoretical frameworks conclude that children develop behaviours and internal cognitive models of both the self and relationships with other people through their primary caregivers. Positive relationships during childhood with adults who provide affection and a secure base for exploring the social environment are fundamental to positive socioemotional development. However, the children with poor quality family relationships raised in socially disadvantaged environments may be at risk of learning negative relationship patterns, thus leading to aggressive behaviour or to them becoming victims of peer aggression [103].

The results of the studies reviewed also underpin the importance of considering the differential susceptibility of children to parenting behaviours. In particular, the evidence suggests that the factors underlying perpetration and victimisation in preschool aggression vary depending on the child’s gender and the parent–child dyads [61,80]. However, not all of the studies analysed the gender differences or include the data on parent–child dyads, making it difficult to draw clear conclusions. In line with Narayana and Naerde [82], future research should shed light on the mechanisms underlying the transmission of this risk from parents to children and examine how this may differ between the mothers and fathers at different developmental stages.

While it was not specifically an objective of this review, as noted in the introduction, another critical question for the researchers and practitioners has been whether it is possible to extend the concept of bullying to the study of aggressive behaviours occurring during early childhood [104,105,106]. Only four (10.25%) of the studies reviewed have used the term “bullying” to refer to aggressive behaviour in preschool [63,68,71,91]. However, only two of them included specific measures of bullying in the description of their method, or they have analysed the repeated nature of such behaviours [68,91]. Nevertheless, both of the studies measured bullying only through the teachers’ reports, and neither of them analysed other peripheral roles such as defenders or included measures to analyse the intentionality, stability and power inequality characterising these behaviours [20]. Nor did they include a definition of bullying to clarify these dimensions for the teacher respondents. It is therefore difficult to arrive at firm conclusions on the relevance of discussing bullying at the preschool stage, and more research is needed. The qualitative research has shown that children, as well as teachers and parents, identify bullying behaviour during childhood. González-Moreno et al. [19], for example, found that from an early age, children are able to identify bullying behaviours and attribute meanings to these behaviours in very similar manners to those of adolescents. However, these researchers note, nonetheless, that such behaviours could be termed ‘proto-bullying’ and identifying them could be key to preventing bullying behaviour at later stages.

### Limitations and Recommendations

Despite the merits of this review, some important limitations do exist, first and foremost, there are those that are related to the search. On the one hand, only articles in English and Spanish were included, thus potentially excluding relevant studies that were published in other languages. Moreover, the use of a limited number of databases and the search terms themselves may have led to the exclusion of some studies addressing the research question posed.

Secondly, the diversity of the studies included in this review means that constructs such as aggressive behaviour itself, parenting styles or certain parenting practices may take on varying meanings in different cultural and social contexts. This makes the comparison difficult, and it means that the results should be treated with caution, taking into account these potential differences. This being the case, it is worth noting that in those studies where the information from parents and teachers was available in relation to the children’s aggressive behaviour, there were differences in the levels of reported aggression. Although engaging a range of informants is important when one is analysing aggressive behaviour, the parents’ and teachers’ reports may be affected by different factors. These include the fact that children behave differently in different environments and with different people. Moreover, aggressive behaviours, such as relational behaviours, may be easier to perceive in a context of ongoing peer relationships, such as in school, as opposed to other contexts outside the home environment, such as parks, playgrounds and so on. Alternatively, the informants may show lower or higher tolerance for aggressive behaviours [83]. We believe that the teachers’ reports may be more descriptive of the aggression in the school environment because of the time they share with their pupils and their knowledge of the relationship dynamics among them. For this reason, greater consideration has been given to the teachers’ reports of aggressive behaviour in order to draw conclusions about the relationship between the family variables and preschool aggression. This is presumably a limitation of this review.

Thirdly, and again, when one is making comparisons and drawing conclusions, it should be borne in mind that the measurement instruments that are used differ from one study to the next. In this regard, the family factors were measured mainly through self-reports by the fathers, mothers or by both of the parents. Self-reports have the advantage of capturing an overall picture of parenting, but they may also be vulnerable to social desirability and recall biases [91]. The literature suggests that supplementing these measures with direct evaluations of parenting is likely to increase the predictive power of these constructs [87]. Alternatively, as suggested by Nelson et al. [61], it would be appropriate to supplement them with the information provided by the children about their parents’ behaviour, and whenever possible, to find out the spouse/partner’s view. The fact is that virtually no studies have been found with child-reported information raises the likelihood of the researchers overlooking some cases of victimisation and perpetration. Finally, the tools used to measure the aggression and family variables differed between the articles, which also limits comparability between the studies and the opportunity to draw more accurate conclusions.

Fourthly, the articles in this review are mostly cross-sectional in design, so any conclusions about the influence of the family factors on aggressive behaviour must acknowledge the difficulties in establishing the causality of the factors. It would be advisable to conduct longitudinal studies over extended periods of time and to plan studies with large sample sizes. This would help in gaining a better understanding of the association between the family factors and preschool aggression. In addition, most of the samples involved middle-class, White participants. The inclusion of comparison groups of families from different socioeconomic backgrounds and cross-cultural studies would permit a more direct examination of the specific effects of parenting culture on the children’s psychosocial development. In this sense, the inconsistencies in certain findings may be partly attributable to the limitations of the analytical approaches and the sample that were used. For example, there has been little control for the other individual, family and contextual factors that mediate or moderate the relationship between the perpetration or victimisation behaviours and the family variables. The future research should try to identify such factors that may play a role in mediating or moderating this relationship.

Nevertheless, in answer to the question that is posed in this review, it is clear that there are family variables related to peer aggression and victimisation in the preschool environment. However, most of the current intervention strategies for the prevention of school-based perpetration of aggression and victimisation neglect the role of family relationships [107]. The findings of the studies contained in this review suggest that intervention programmes aimed at reducing the children’s personal risk factors should be combined with counselling and training programmes for the parents. In general, training programmes can help the parents to develop healthier relationship patterns with their children, with the goal of making the parents aware that their relationship with their child is the basis for the child’s psychosocial development [87]. The programmes should aim to promote positive parenting practices, foster the children’s perceived support and affection, and establish patterns promoting intrafamilial communication and closeness [108]. Not only should such interventions aim to promote positive parenting skills, but they should also help the parents to deal with problems in their personal lives, including medical care, stress, job training, developing communication skills and identifying available community resources [109], and seeking support from teachers [110].

Furthermore, the existing research suggests that the outcomes of aggressive behaviour prevention programmes in the school environment could be improved by focusing on the at-risk groups identified in preschools, and not only by targeting primary and secondary schools [71]. In line with Paschall et al. [85], we further recommend that intervention programmes incorporate specific additional support for the parents who face sociodemographic risk factors linked to adverse social and economic conditions, and who are exposed to other harmful events such as domestic abuse, criminality, psychopathology and so on. The types of preventive interventions that appear to be most effective for the at-risk groups are those that start early and are holistic, involving a systemic approach with multiple interventions that touch on the individual, family and community levels [109].

## 5. Conclusions

This scoping review shows that different family environmental factors increase the risk of developing perpetration and victimisation behaviours in the preschool setting. However, the relationship between the family variables and aggression in preschool children is complex, and it may be mediated by other co-existing factors, such as gender, teacher closeness and the parent–child dyads, or personal factors such as theory of mind and the children’s effortful control. The results have important implications for prevention and intervention programmes to prevent aggression from spilling over into later educational stages.

## Figures and Tables

**Table 1:** Search terms used.

Search Terms
1. (“preschool *” OR “preschool children” OR “preschool-aged children” OR “early child*” OR “2–6 yrs”).ti
2. (“victim *” OR “perpetrat *” OR “aggress *” OR “bullying” OR “bully” OR “bully/victim” OR “peer aggress *” OR “peer violen *” OR “peer victim *” OR “peer abus *”).ti
3. (“family” OR “families” OR “family context” OR “parent *” OR “mother” OR “father”).ti
4. Searches 1, 2 and 3 were performed in each database.

**Table 2:** Summary of the inclusion and exclusion criteria.

Inclusion	Exclusion
Qualitative and quantitative research published in peer-reviewed journals.	Articles describing interventions or prevention programmes, literature reviews, systematic reviews, conference papers, doctoral theses and newspaper articles.
Participants aged two to six years old.	Participants who were less than two or more than six years old.
Participants in the study had to belong to general populations.	Clinical samples or subgroups.
Research analysing aggressive peer interactions in school environments.	Research analysing aggressive interactions beyond peer relationships in school settings.
Research examining the relationship of preschool aggression using family or parental variables as predictor variables.	Research examining the relationship of preschool aggression not using family or parental variables as predictor variables.
Published in English and Spanish.	Published in languages other than English and Spanish.

## Data Availability

All data generated as part of this study are included in the article.

## Appendix A

**Table A1:** Analysis of the selected studies (*n* = 39).

Authors (Year) Country	Sample and Main Characteristics	Aim of the Study	Study Design and Methodology Approach	Types of Preschool Aggression Examined	Preschool Aggression Assessment Tools	Family Variables Included	Statistical Analyses Included to Test the Association between Preschool Aggression and Family Variables	Key Findings about the Relationship between Preschool Aggression and Family Variables
DeMulder, Denham, Schmidt and Mitchell [84]. United States.	94 children and their mothers Children’s sex: 51 boys, 43 girls Age range: 38–58 months Ethnicity: 81% White	Investigate the relationships between preschoolers’ secure-base behavior at home, stressful family conditions and relationships with peers and teachers in preschool.	Cross-sectional	Anger/Aggression (perpetration)	“Social Competence and Behavior Evaluation”- Teacher reports (SCBE-30; LaFreniere and Dumas, 1996).	Security with mother, assessed through observations at home (AQS, Waters et al., 1994). Family stress, self-reported by mothers through the “Life Experiences Survey” (LES, Sarason et al., 1978).	Bivariate and multivariate analysis	Boys and girls less securely attached to their mothers expressed more anger/aggression in preschool. Higher levels of family stress were only significantly related to anger/aggression for boys.
Honig and Su [67]. Taiwan.	90 children: 30 in mother’s custody, 30 in father’s custody, and 30 from two-parent families Children’s sex: 50% girls Mean age reported = 5.39 (SD = 1.00) Ethnicity: not reported	Examine the effects of divorce and custody arrangements on preschool children’s emotional adjustment.	Cross-sectional	Hostile, aggressive behavior(perpetration)	“Preschool Behavior Questionnaire”-Teacher reports (PBQ; Behar and Stringfield, 1974).	Effect of divorce and custody arrangements assessed through open-ended interviews with parents.	Bivariate and multivariate analysis	No significant differences were found between children from married families in comparison with children living in divorced families. Boys are more aggressive than females, regardless of family configuration. Children who were in custody with same-sex parents had significantly lower aggression scores.
Farver, Xu, Eppe, Fernandez and Schwartz [77]. United States.	431 children, their mothers and their teachers Children’s sex: 50% girls Age range: 43–59 months Ethnicity: 85% African American; 15% Latino	Analyze relations among family conflict, community violence, mothers depressive symptoms and children’s socioemotional functioning.	Cross-sectional	Composite measure of aggression, including physical, verbal, relational and bullying (perpetration)	“Social Behavior Rating Scale”-Teacher reports (SBRS, Schwartz, 2000).	Family conflict, assessed through mothers’ scores on the Conflict Tactics Scale (Strauss, 1979). Mothers’ depressive symptoms, assessed through scores on the Beck Depression Inventory (Beck et al., 1996).	Bivariate and Multivariate analysis Mediational path analysis: Mothers’ depressive symptoms y children’s social awareness were tested as mediators	Mothers’ reports of family conflict were not correlated with teachers’ ratings of aggressive behaviors.
Ngee Sim and Ping Ong [80]. Singapore.	286 children, their parents and 35 teachers Children’s sex: 50% girls Age range: 4–6 years old Ethnicity: 100% Singapore Chinese	Examine the relationships between two forms physical punishment (caning and slapping) and child aggression, testing for moderation by authoritative control and rejection.	Cross-sectional	Composite measure of aggression, including physical, verbal, relational and bullying (perpetration)	Teacher reports of child aggression through items adapted and expanded from Dodge and Coie (1987).	Physical punishment and trough parents’ reports using the ”Conflict Tactics Scale” (Strauss, 1990). Authoritative control assessed through mothers’ and fathers’ reports using the Parental Authority Questionnaire (Buri, 1991). Perceived parental rejection through structured interviews with children using items from the “Parental Acceptance-Rejection Questionnaire” (Rohner, 1990).	Bivariate and Multivariate analysis Moderation analyses conducted Authoritative control and perceived parental rejection were tested as moderators	Relations were found between each punishment type and aggression depending on the specific parent–child dyad. Father caning is related to aggression, regardless of child gender, whereas mother caning is related to child aggression only at low children’s perceived rejection. Mother slapping is related to sons’ aggression, whereas father slapping is related to daughters’ aggression only at low children’s’ perceived rejection values. Authoritative control did not play a moderate role.
Casas et al. [86]. United States.	122 children and their parents (119 mothers and 85 fathers) Children’s sex: 57% girls Age range: 2 years and 6 months old to 5 years and 10 months old Ethnicity: 87% Anglo American	Analyse how children’s use of relational and physical aggression varies according to parent–child relationships.	Cross-sectional	Physical and relational aggression (perpetration)	Teachers’ reports through the “Preschool Social Behavior Scale” (Crick et al., 1997). Parents’ reports through the “Children’s Social Experiences measure” (CSE, Crick, Casas et al., 1999).	Parental reports of parental styles using the “Parenting Practices Questionnaire” (PPQ, Robinson et al., 2001), psychological control using the “Psychological Control Measure (Olsen et al., 2022), attachment styles using the “Parent/Child Reunion Inventory (Marcus, 1991).	Bivariate and Multivariate analysis	Authoritarian and permissive parenting styles were positively related to children’s relational aggression. Authoritative parenting style was associated with less children’s physical aggression. Parental psychological control was related with relational and physical aggression. Insecure attachment was associated with relational and physical aggression. Associations varies according to the sex composition of the parent–child dyad.
Nelson et al. [89]. China.	215 children and their parents (180 mothers and 167 fathers) Children’s sex: 53% girls Age range: 46–76 months Ethnicity: not reported	Assess the combined and differential contributions of spouse reported parenting styles in physical and relational aggression.	Cross-sectional	Physical and relational aggression (perpetration)	Peer nomination procedure through the adaptation of the items from Crick, Casas and Mosher (1997).	Negative parenting (coercion and psychological control) assessed through parent’s reports of their partners practices using items adapted from previous measures (Robinson et al., 2001; Yang et al., 2004).	Bivariate and Multivariate analysis	Combined parenting effects were more prevalent than differential effects in predicting aggression. Physical coercion was predictive of relational and physical aggression in boys, and psychological control was primarily associated with physical and relational aggression in girls.
Ostrov, Crick and Stauffacher [11]. United States.	50 children (25 sibling dyads, 13 same-sex and 11 mixed-sex pairs) Children’s sex: 48% girls Age range: 27–61 months Ethnicity: 72% Europena American	Examine the relationship between the sibling relationship and school-based peer aggression.	Short-term longitudinal	Physical and relational aggression (perpetration)	Naturalistic observations in classrooms at two different points (fall and spring).	Sibling physical and relational aggressive behavior through naturalistic observations in classrooms.	Bivariate and Multivariate analyses	Older sibling’s relational and physical aggression predicted younger sibling’s relational and physical aggression with peers.
Ostrov and Bishop [76]. United States.	47 children and their parents (43 mothers and 4 fathers) Children’s sex: 63% girls Age range not reported Mean age reported = 43.54 months (SD = 8.02) Ethnicity: 63.8% Caucasian	Investigate the relationships between parent–child relationships qualities and physical and relational aggression with peers at school.	Cross-sectional	Physical and relational aggression (perpetration)	Naturalistic observations in classrooms. Teachers’ reports through the “Preschool Social Behavior Scale” (Crick et al., 1997). Parents’ reports through the “Children’s Social Behavior” scale (CSB, Crick, 2006a).	Parent–child conflict and child’s use of physical aggression toward the parent, assessed through parent’s reports using the “Parent Qualities Measure (PQM, Crick, 2006b).	Bivariate and Multivariate analysis	Parent–child conflict was associated with relational aggression, even when controlling for physical aggression and gender.
Shin and Kim [90]. South Korea.	297 children, their parents, and teachers (data about number of parents and teachers not reported) Children’s sex: 44.1% girls Age range: 4–5 years old Ethnicity: not reported	Examine relationships between child characteristics (aggression and withdrawal), parenting behaviors, teacher–child relationships and peer victimization.	Cross-sectional	Relational and physical victimization Aggression and withdrawal (child characteristics)	Teachers’ reports about peer victimization based on previous studies (Schwartz et al., 2002). Teachers’ reports about aggression and withdrawal through the “Social Competence and Behaviour Evaluation” scale (SCBE, LaFreniere and Dumas, 1996).	Parenting behaviours (warmth, neglect/rejection, physical coercion), assessed through parents’ report on an adapted measure (Kim and Park, 2006).	Bivariate and Multivariate analysis	Parental neglect/rejection increased the probability of being victimised by peers. Children who were characterised by withdrawal or aggressive behaviour were likely to be victimised by peers.
Amin, Behalik and El Soreety [72]. Egypt.	50 children and their mothers Children’s sex: 50% girls Age range: 3–6 years old Ethnicity: not reported	Analyze the prevalence and factors associated with aggression in preschool settings.	Cross-sectional	Composite measure of aggression, including physical, verbal, and relational forms (perpetration)	Naturalistic observations in classrooms.	Mothers’ education and occupation (method of assessment not reported).	Bivariate analyses	Aggressive scores were lower among children whose mothers had university education. However, aggressive scores were higher among children whose mothers work out of home.
Buyse, Verschueren and Doumen [93]. Belgium.	127 children, their mothers, and teachers (number of teachers not reported) Children’s sex: 50.3% girls Age range not reported Mean age reported = 4.11 years old (SD = 4) Ethnicity: not reported	Evaluate the moderating role of teacher–child closeness for the association between mother-child attachment quality.	Cross-sectional	Composite measure of aggression (perpetration)	Teachers’ reports through the aggressive behavior subscale of the “Child Behavior Scale” (CBS, Ladd and Profilet, 1996).	Mother-child attachment assessed through a semi-structured observation in the home environment.	Bivariate and Multivariate analysis Moderation analyses conducted Teacher–Child Closeness was tested as moderator	Children with lower quality attachment to their mother showed higher scores of aggressive behaviours. Teacher–child closeness moderates the relationships between lower quality of attachment and aggressive behaviour.
Olson et al. [79]. United States.	199 children, their mothers, and teachers (number of teachers not reported) Children’s sex: 59.2% girls Age range = 32–45 months Ethnicity: 91% European American heritage	Identify preschool-age self-regulatory, social-cognitive and parenting precursors of children’s peer aggression following the transition to school.	Longitudinal	Composite measure of aggression, including physical, verbal, and object aggression forms (perpetration)	Naturalistic observations in classrooms at two different points (at age 3 and 6 years). Teachers ratings at 3 years using the “Caregiver/Teacher Report Form” (Achenbach, 1997). Teacher ratings at 6 years using the “Inventory of Peer Relations” (Dodge and Coie, 1987).	Parenting risk (corporal punishment and low warmth/responsiveness) assessed using interview-based and questionnaire (Parenting Dimensions Inventory; Power, 1993).	Bivariate and Multivariate analyses Interaction analyses conducted between the study variables	Children showing high levels of aggressive peer interaction in school also showed higher levels of adverse parenting than others did. Early corporal punishment was associated with higher peer aggression across the transition from preschool to school. Regarding interaction, low warm responsiveness was the best precursors of later peer aggression in children with low levels of theory of mind.
Jansen et al. [68]. Netherlands.	6376 children, their parents, and teachers (number mother, parents and teachers not reported) Children’s sex: 49% girls Age range = 5–6 years old Ethnicity: 57% Dutch	Examine the prevalence and socioeconomic disparities in bullying behaviour among young elementary school children.	Cross-sectional	Bullying, including physical, verbal, relational and material forms (perpetration and victimization)	Teachers reports of bullying by means of questionnaires (Perren and Alsaker, 2006).	Family socioeconomic status assessed by a parental report in an ad hoc questionnaire.	Bivariate and Multivariate analyses	Children from socioeconomically disadvantaged families had a particular risk of involvement as bully or bully victim. Parental education level was the only indicative of socioeconomic status associated with victimization.
Katsurada [63]. Japan.	175 children, their parents, and teachers (175 mothers, 124 fathers, while teachers were not reported) Children’s sex: 45% girls Age range not reported Mean age reported = 62.24 months (SD = 5.05) Ethnicity: not reported	Examine the relationships between parents’ physical affection and children behavior in preschool settings.	Cross-sectional	Hostile, aggressive behavior (perpetration)	“Preschool Behavior Questionnaire”, Teacher report (PBQ; Behar and Stringfield, 1974).	Parental physical affection assessed by mothers’ and father’s reports in an ad hoc questionnaire.	Bivariate and Multivariate analysis	Children who received more physical affection from mothers or fathers were less likely to be aggressive at preschool. Only fathers’ physical affection was related with less aggressive behaviours when mothers’ physical affection was controlled.
Seçer, Gülay Ogerlman, Önder and Berengi [94]. Turkey.	200 children, their mothers, and their teachers (number of teachers not reported) Children’s sex: 45% girls Age range = 5–6 years old Ethnicity: not reported	Investigate the relationship between mothers’ self-efficacy perception toward parenting and peer relations in school settings.	Cross-sectional	Composite measure of aggression (perpetration) Composite measure of peer victimization	Teachers’ reports through the aggressive behavior subscale of the “Child Behavior Scale” (CBS, Ladd and Profilet, 1996). Teachers’ reports through the “Peer Victimization Scale (Ladd and Kochenderfer-Ladd, 2002).	Parenting self-efficacy perception assessed by mothers’ responses to the “Parenting Sense of Competence Scale” (Gibaud-Wallston and Wandersman, 1978).	Bivariate and Multivariate analysis	Low parenting self-efficacy perception among mothers was associated with higher levels of peer aggression and peer victimization in schools’ settings.
Hammes, Crepaldi and Bigras [75]. Canada.	278 children, their families, and teachers (79 teachers; the number of mothers and fathers not reported) Children’s sex: 50.7% girls Age range not reported. Mean age reported = 5.6 years old Ethnicity: not reported	Test the association between family functioning of preschoolers and their socioaffective competencies at the end of the first grade.	Short-term longitudinal	Anger/Aggression (perpetration)	Teachers reports at two different points (at age 5 and 7 years), through the “Social Competence and Behavior Evaluation”, Teacher report (SCBE-30; LaFreniere and Dumas, 1996).	Family functioning reported by parents through the “Self-Report Family Inventory” (SFI; Beavers and Hampson, 1990).	Bivariate and Multivariate analysis	Children with higher family harmony (less conflicting family relationships in the preschool period), display less aggressive behaviors in school settings during first grade.
Ziv [74]. United States.	256 children, their families (242 mothers, and 14 grandmothers) and teachers number not reported) Children’s sex: 50.7% girls Age range = 48–63 months old Ethnicity: 43% black, 37% white, 11% Asian, 9% Latino	Examine the links between exposure to violence in the family/home and maladjusted behaviour in preschool.	Short-term longitudinal	Composite measure of aggression (perpetration)	Teachers reports at two different points (three months apart), through the problem behavior scale used in the ACYF (2006) study.	Violence exposure in the family/home reported by mothers through the same measure used in the Family and Child Experiences Study (ACYF, 2006).	Bivariate and Multivariate analysis Mediational path analysis Children’s’ social information process was tested as mediators	Children who reported witnessing and/or experiencing violence were more likely to behave aggressively with peers in the school setting. Children exposed to violence behave aggressively, at least in part, because they believe that aggression is appropriate in challenging peer situations.
Bigras and Crepaldi [96]. Canada.	278 children, their families (217 mothers and 172 fathers), and teachers (*n* = 151) Children’s sex: not reported Age range not reported Mean age reported = 68.02 months old Ethnicity: not reported	Clarify the relationships between parental values and children’s social behaviours.	Cross-sectional	Anger/Aggression (perpetration)	Teachers reports through the “Social Competence and Behavior Evaluation”- Teacher report (SCBE-30; LaFreniere and Dumas, 1996).	Parental values (individualism, collectivism and verticalism) reported by parents through an ad hoc questionnaire.	Bivariate and Multivariate analysis	Combinations of individualistic and collectivistic values (more observed in mothers that in parents), were associated with greater social competence in children and, therefore, less aggressive behaviours.
Jung, Raikes and Chazan-Cohen [83]. United States.	914 children, their mothers and teachers (914 mother-teacher dyads) Children’s sex: 50.2% girls. Age range = 36–73 months old. Ethnicity: 38.1% European-American; 34% African American; 24.5 Hispanic American.	Compare behaviour problems of children of mothers with elevated depressive symptoms and non-elevated depressive symptoms.	Cross-sectional	Composite measure of aggression (perpetration)	Mothers’ and teachers reports through the “Family and Child Experiences Survey (FACES) interview” (Administration on Children, Youth, and Families, 1998).	Maternal depression reported by mothers through the “Center for Epidemiologic Studies Depression Scales” (CES-D-SF, Ross et al. 1983).	Bivariate and Multivariate analysis	Children of mothers with non-elevated depressive symptoms were reported to have more aggressive behaviors than children of mother with non-elevated depressive symptoms by their mothers’ rating of aggression. However, there were no differences in teachers’ rating of aggressive behaviors.
Meysamie et al. [65]. Iran.	1403 children, their parents, and teachers (number mother, parents and teachers not reported) Children’s sex: 48% girls Age range = 3–6 years old Ethnicity: 38.1% Eurpean-American; 34% African American; 24.5 Hispanic-American	Estimate the prevalence and associated factors of childhood aggression.	Cross-sectional	Physical, verbal and relational aggression forms (perpetration)	Parents’ and teachers’ reports through the “Aggression Scale” developed by Shahim (2006).	Occupation and level of education of parents, history of smoking in mother during pregnancy, number of siblings, living with one or both parents, chronic disease in family and death of child relatives, reported by parents and teachers through an ad hoc questionnaire.	Bivariate and Multivariate analyses	Results differ according to parents’ and teachers’ reports. Focusing on teachers’ reports of aggression in school settings, physical aggression was not associated with any family factor examined. Verbal aggression was associated with chronic disease in family members, and defeat of a family member. Relational aggression was associated with chronic disease in family members and maternal occupational status (employee).
Nelson et al. [61]. Russia.	207 children, their parents, and teachers (204 mothers, 164 fathers, number of teachers not reported) Children’s sex: 52,1% girls Age range not reported Mean age reported = 5.1 years old (SD = 0.72) Ethnicity: not reported	Examine the relationship between parental psychological control and childhood physical and relational aggression.	Cross-sectional	Physical and relational aggression forms (perpetration)	Teachers’ reports through the “Preschool Social Behavior Scale” (Crick et al., 1997).	Psychological control dimensions (shaming/disappointment; constraining verbal expression; invalidating feelings, love withdrawal; guilt induction) reported by partners using items derived from the “Parental Psychological Control” scale (PPC, Hart and Robinson, 1995).	Bivariate and Multivariate analyses	All dimensions of psychological control, except for invalidating feelings, were associated with physical and relational aggression, although predominantly in same-sex parent–child dyads.
Seçer, Gülay Ogerlman and Önder [95]. Turkey.	200 children, their fathers, and teachers (number of teachers not reported) Children’s sex: 48% girls Age range = 5–6 years old Ethnicity: not reported	Investigate the relationship between fathers’ self-efficacy perception toward parenting and peer relations in school settings.	Cross-sectional	Composite measure of aggression (perpetration) Composite measure of peer victimization	Teachers’ reports through the aggressive behavior subscale of the “Child Behavior Scale” (CBS, Ladd and Profilet, 1996). Teachers’ reports through the “Peer Victimization Scale (Ladd and Kochenderfer-Ladd, 2002).	Parenting self-efficacy perception assessed by fathers’ responses to the “Parenting Sense of Competence Scale” (Gibaud-Wallston and Wandersman, 1978).	Bivariate and Multivariate analyses	Low parenting self-efficacy perception among fathers was associated with higher levels of peer aggression and peer victimization in schools settings. This relationship was stronger between fathers’ self-efficacy and peer victimization.
Jia, Wang and Shi [66]. China.	1164 children, their parents, and teachers (53 teachers) Children’s sex: 45.1% girls Age range = 3–6 years old Ethnicity: not reported	Examine the relationship between parenting and proactive versus reactive aggression in preschool.	Cross-sectional	Proactive and reactive aggression (perpetration)	Teachers’ reports through the “Aggressive Behavior-Teacher’s Checklist” (Dodge and Coie, 1987).	Parenting behavior covering two type of practices (supportive/engaged and hostile/coercive) assessed by parent’s responses to the “Parent Behavior Inventory” (PBI, Lovejoy et al., 1999). Family characteristics: family structure, household income, education and occupation (self-reported by parents).	Bivariate and Multivariate analyses	Hostile/coercive parenting and less educated fathers were independent risk factors of both proactive and reactive aggression in the preschool setting.
Werner et al. [97]. United States.	175 children, their mothers, and teachers (11 teachers) Children’s sex: 52% girls Age range not reported. Mean age reported = 4.30 years (SD = 0.62) Ethnicity: 85% white	Evaluate whether the quality of mothers’ conversation (coaching qualities) with preschoolers impacts the development of relational aggression in school.	Short-term longitudinal	Relational and physical aggression (perpetration)	Teachers reports at two different points (twelve months apart), through the “Preschool Social Behavior Scale” (Crick et al., 1997).	Social Coaching Qualities (elaboration, emotion references and rule violation) assessed through coding of mothers’ conversations with children during a coaching task in a laboratory session.	Bivariate and Multivariate analyses	After controlling for physical aggression, children whose mothers used average or high levels of elaborative, emotion-focuses social coaching abut relational conflicts were less relationally aggressive the following year.
Paschall et al. [85]. United States.	1101 children, their mothers and teachers (number of teachers not reported Children’s sex: 50% girls Age range or Mean age not reported Ethnicity: 36% European American, 34.7% African American, 24.6% Hispanic American	Examine how maternal parenting behaviors at 3 years old were associated with children’s classroom aggression in pre-kindergarten.	Longitudinal	Composite measure of physical and verbal aggression (perpetration)	Teacher reports of aggressive behavior through the “Teacher Report Form of the Achenbach Child Behavior Checklist (TRF, Achenbach, 1991).	Maternal behaviors (supportiveness, negative regard, intrusiveness and detachment) were observationally assessed during a “Three-Bag” 10 min videotaped mother-child interaction task at 3-year-old child (Love et al., 2005).	Bivariate and Multivariate analyses	Children exposed to detached parenting at 3 years old showed higher levels of classroom aggression in pre-kindergarten than children exposed to supportiveness parenting.
Jiménez et al. [103]. United States.	1007 children, their mothers, and teachers (number of teachers not reported) Children’s sex: 51% girls Age range not reported Mean age reported = 61 months old Ethnicity: 46% African American, 36% White, 24% Latino	Examine relationships between adverse childhood experiences in early childhood and teacher reported behavioral problems in preschool.	Longitudinal	Composite measure of aggression (perpetration)	Teacher reports of aggressive behavior through the “Teacher Report Form of the Achenbach Child Behavior Checklist (TRF, Achenbach, 1991).	Adverse childhood experiences (child maltreatment, parental substance use incarceration, caregiver treated violently) assessed through maternal reports at 5 years follow-up interview with ad hoc interview script, before conducting the teachers interviews about aggression.	Bivariate and Multivariate analyses	Children who suffered three or more adverse childhood experiences were at higher risk of being involved in classroom aggression perpetration.
Narayana and Naerde [82]. Norway.	1007 children, their mothers, and teachers (number of teachers not reported) Children’s sex: 51% girls Age range not reported. Mean age reported = 61 months old Ethnicity: 46% African American, 36% White, 24% Latino	Analyze the associations between parental depressive symptoms and child behavior problems in preschool.	Longitudinal	Composite measure of aggression (perpetration)	Mothers and Fathers reports of aggressive behavior on the Child Behavior Checklist (CBCL) and teachers’ reports of aggressive behavior through the “Teacher Report Form of the Achenbach Child Behavior Checklist” (C_TRF, Achenbach and Rescolar, 2000), at 48 months after childbirth.	Parental depressive symptoms at 6, 12, 24, 36 and 48 months after childbirth, assessed through the Hopkins Symptom Checklist (SCL-10).	Bivariate and Multivariate analyses	Mothers’ depressive symptoms at 6 months after childbirth predicted aggressive behaviors at 48 months after childbirth, while fathers’ did not. Increase in paternal depression symptoms from 6 to 48 months were associated with more aggressive problems, while a corresponding maternal rise over the months did not predict aggressive behavior.
Baker, Jensen and Tisak [69]. United States.	143 children, their parents, and teachers (number not reported) Children’s sex: 49.6% girls Age range not reported Mean age reported = 51.7 months (SD = 8.52) Ethnicity: 84.8% Caucasian	Test relations between proactive and reactive aggression, executive function and single-parent status.	Cross-sectional	Proactive physical/relational and reactive physical/relational aggression (perpetration)	Teachers’ reports through the “Preschool Proactive and Reactive Aggression-Teacher Repot” (PPRA-TR, Ostrov and Crick, 2007).	Single-parent status self-reported by parents.	Bivariate and Multivariate analyses	Children of single parents exhibit greater levels of both types of relational aggression (proactive and reactive), and reactive-physical aggression.
Matheson et al. [78]. Australia.	69116 children, and teachers (number not reported) Children’s sex: 49.5% girls Age range not reported. Mean age reported = 5.6 years old (SD = 0.4) Ethnicity: not reported	Establish the independent and moderating effect of childhood maltreatment and parental schizophrenia disorders on early childhood social-emotional functioning.	Longitudinal	Composite measure of aggression including physical aggression, bullying, among others (perpetration)	Teachers’ reports through the “Australian Early Development Census” (AEDC, Australian Government, 2009) at children’s aged 5 years old.	Child maltreatment and parental schizophrenia disorders assessed by government records.	Bivariate and Multivariate analyses	Childhood maltreatment and parental schizophrenia disorders were related independently with higher aggressive behaviors in preschool. However, childhood maltreatment effect of aggressive behavior is greater in comparison to parental schizophrenia disorders.
Tzoumakis et al. [81]. Australia.	69116 children, and teachers (number not reported) Children’s sex: 49.5% girls Age range not reported Mean age reported = 5.6 years old (SD = 0.4) Ethnicity: not reported	Analyze the impact of parental criminal offending on preschool aggression at age 5 years old.	Longitudinal	Composite measure of aggression including physical aggression, bullying, among others (perpetration)	Teachers’ reports through the “Australian Early Development Census” (AEDC, Australian Government, 2009) at children’s aged 5 years old.	Maternal and paternal criminal offending assessed by government records.	Bivariate and Multivariate analyses	Maternal and paternal criminal offending was associated with high levels of aggression at 5 years old. Strength of the relationship was greater when parents were involved frequently in offences.
Baker et al. [70]. United States.	89 children, their parents, and teachers (number not reported) Children’s sex: 48.5% girls Age range = 3–5 years old Mean age reported = 51.27 months (SD = 7.77) Ethnicity: not reported	Explore the associations between family socioeconomic level, theory of mind and aggressive behavior in preschool.	Cross-sectional	Relational and physical aggression (perpetration)	Teachers reports through the “Preschool Social Behavior Scale” (Crick et al., 1997).	Socioeconomic status reported by parents.	Bivariate and Multivariate analyses Moderation analyses conducted: Theory of mind tested as moderator	Low socioeconomic status was related with higher scores on relational aggression. However, theory of mind moderated this relationships and children with high levels of theory of mind did not receive higher scores on relational aggression regardless the socioeconomic status of their families.
Kokanović and Opić [64]. Croatia	669 children, their parents and teachers (numbers not reported) Children’s sex: 50.3% girls Age range = not reported Mean age = not reported Ethnicity: not reported	Study differences in aggressive and prosocial behaviours among preschool children in relation to family differences.	Cross-sectional	Composite measure of aggression (perpetration)	Teachers reports through the “Aggressive and Prosocial Behavior in Preschool Children” (PROS/AG, Žužul and Vlahović- Štetić, 1992)	Parents’ reports of sociodemographic data (family roles, number of siblings and single-parent status).	Bivariate and Multivariate analyses	There were no differences in aggressive behaviours among only children and children with siblings, and also between single-parent and two-parent families.
Navarro et al. [71]. Spain.	577 children, their parents (mothers, 67.6%; fathers, 7.6%; both, 24.8%) and teachers (*n* = 577) Children’s sex: 49.9% girls Age = 5 years old, in the last assessment Ethnicity: not reported	Identify early individual and family risk factors (at age 3) of being peer victimised during preschool (ages of 4 and 5).	Longitudinal	Composite measure of peer victimization	Parents’ and teachers’ reports at age 4 and 5 through the “Strengths and Difficulties Questionnaire” (SDQ, Goodman, 1997).	Parents’ reports at age 3 of problems during pregnancy (emotional problems, conflict, economic problems), history of abuse, parental practices (APQ-Pr, de la Osa et al., 2014), psychopathology (ASR, Achenbach and Rescorla, 2003).	Bivariate and Multivariate analyses	Low socioeconomic status family, and parental practices characterised by lower norms at age 3 increased the risk of parents and teachers reporting persistent victimization at age 4 and 5.
Güngör et al. [99]. Turkey.	90 children, their father (*n* = 90), and teachers (*n* = 35). Children’s sex: 46.7% girls Age range = 5–6 years old Mean age = not reported Ethnicity: not reported	Examine the relationships between peer aggression in preschool and fathers’ duration of work and time spent with children.	Cross-sectional	Composite measure of aggression (perpetration)	Teachers’ reports through the aggressive behavior subscale of the “Child Behavior Scale” (CBS, Ladd and Profilet, 1996).	Fathers’ reports of educational background, occupation, working hour during weekdays and weekends, number of working days, duration of spending time with the child and activities do together assessed by an ad hoc form.	Bivariate and Multivariate analyses	Long working hours of fathers, decreased free days in a week, and not spending time with children increased the likelihood that children behave aggressively with their peers at school.
Yu et al., [91]. United States.	133 children and their mothers (*n* = 133), and teachers (*n* = 35) Children’s sex: 47% girls Age range = not reported Mean age = 4.48 (SD = 0.91) Ethnicity: 100% Chinese American	Analyse the effects of two psychological control dimensions (love withdrawal and guilt induction) on children’s bullying behavior in preschool.	Short-term Longitudinal	Composite measure of bullying, including physical, verbal and relational bullying (perpetration)	Teachers reports at two different points (six months apart), through the “Teachers Rating Scale” (Hart and Robinson, 1996).	Mothers’ report of low withdrawal and guild induction assessed through the ”Psychological Control Measure” (Olsen et al., 2002).	Bivariate and Multivariate analyses	Maternal love withdrawal predicted more bullying aggressive behavior, whereas guild induction predicted less preschool bullying 6 months later.
Mizokawa and Hamana [98]. Japan	51 children and their mothers (*n* = 44), and teachers (*n* = 8) Children’s sex: 54.9% girls Age range = 58.71 months old Mean age = 65.14 months (SD = 3.73) Ethnicity: not reported	Examine the association between theory of mind, maternal emotional expressiveness and preschoolers’ aggressive behaviors.	Cross-sectional	Physical and relational aggression (perpetration)	Teachers’ reports through the “Preschool Social Behavior Scale” (Crick et al., 1997).	Mothers’ reports of positive and negative emotion towards their children assessed through a measure of self-expressiveness in mother-child relationship (Mizokawa, 2013).	Bivariate and Multivariate analyses Interact in analyses conducted with gender and theory mind	Boys with higher levels of theory of mind and mother showing high negative emotional expressiveness, shower higher relational aggression in the preschool setting. No effect was found for girls.
Ziv and Arbel [87]. Israel.	115 children and their parents, and their teacher (number of teachers not reported) Children’s sex: 53.9% girls Age range = not reported Mean age = 68,5 months (SD = 6.04) Ethnicity: not reported	Study whether there are differences between mothers’ and fathers’ parenting styles and their children’s social difficulties in preschool settings.	Cross-sectional	Author did not specifically analyse aggressive behaviors in school. Perpetration was included in a composite measure of socioemotional difficulties (including emotional difficulties, conduct problems, hyperactivity/inattention and peer relationship problems)	Teachers’ reports through the “Strengths and Difficulties Questionnaire” (SDQ, Goodman, 1997).	Mothers’ and fathers’ reports of parenting styles (authoritative, authoritarian and permissive) assessed through the ”Parenting styles and dimensions questionnaire (Robinson et al., 2001).	Bivariate and Multivariate analyses Mediational path analysis: Aggressive decision-making process was tested as mediator.	Both parents’ authoritative parenting style was associated with less social difficulties in preschool. Association between fathers authoritative parenting style and child social difficulties was not significant after aggressive decision-making process was entered into the equation.
Lau and Williams [88]. Hong Kong.	168 children and their parents (158 mothers, 154 fathers), and their teacher (*n* = 167) Children’s sex: 52% girls Age range = 4–5 years old Mean age = 61 months (SD = 5.51) Ethnicity: 100% Hong Kong Chinese	Explore associations among emotional regulation in mothers and fathers and preschool children’s physical and relational aggression.	Short-term Longitudinal	Physical and relational aggression forms (perpetration)	Teachers’ reports at two different points (six months apart) through the “Preschool Social Behavior Scale” (Crick et al., 1997).	Parents’ repots of emotional regulation (reappraisal, suppression) assessed through the “Emotional Regulation Questionnaire” (Gross and John, 2003).	Bivariate and Multivariate analyses Moderation analyses conducted: Child sex was tested as moderator	Higher levels of reappraisal and lower levels of suppression by mother were associated with higher child relational aggression. There were not significant association with fathers’ emotional regulation or moderation effect by child’s sex.
Lau, Chang and Casas [92]. Hong Kong.	168 children and their parents (158 mothers, 154 fathers), and their teacher (*n* = 167) Children’s sex: 52% girls Age range = 4–5 years old Mean age = 61 months (SD = 5.51) Ethnicity: 100% Hong Kong Chinese	Analyse relationships between mothers’ and fathers’ physical coercion and psychological control and preschoolers’ use of physical and relational aggression in the school setting.	Short-term Longitudinal	Physical and relational aggression forms (perpetration)	Teachers’, mothers’ and fathers’ reports at two different points (six months apart) through the “Preschool Social Behavior Scale” (Crick et al., 1997).	Self-reports and partners’ reports of psychological control using items of the “Psychological Control Scale” adapted from previous measures (Nelson et al., 2013). Self-reports and partners’ reports of physical coercion using items derived from the “the “Parental Psychological Control” scale (PPC, Hart and Robinson, 1995).	Bivariate and Multivariate analyses Moderation analyses conducted: Children’s effortful control was tested as a moderator	Mothers’ and fathers’ physical coercion predicted children’s physical aggression and relational aggression, except fathers’ physical coercion which was unrelated to children’s relational aggression. Child effortful control moderated the effects of fathers’ (but not mothers’) physical coercion on child physical aggression and relational aggression.
